# Supplementary material for: A Bit Stickier, a Bit Slower, a Lot Stiffer: Specific vs. Nonspecific Binding of Gal4 to DNA
Source: Int J Mol Sci. 2021 Apr 7;22(8):3813. doi: 10.3390/ijms22083813 (PMC8067546; doi:10.3390/ijms22083813)
Supplement: Supplementary file 1 [file ijms-22-03813-s001.pdf]

Supporting Information for

A bit stickier, a bit slower, a lot stiffer: specific vs. nonspecific binding of Gal4 to DNA

Thomas Carzaniga[1], Giuliano Zanchetta[1,*], Elisa Frezza[2], Luca Casiraghi[1], Luka Vanjur[1], Giovanni Nava[1], Giovanni Tagliabue[3], Giorgio Dieci[4], Marco Buscaglia[1,*], and Tommaso Bellini[1,*]

[1] Dipartimento di Biotecnologie Mediche e Medicina Traslazionale, Università di Milano, 20054, Segrate (MI), Italy
[2] Université de Paris, CiTCoM, CNRS, F-75006 Paris, France
[3] Proxentia S.r.l., Viale Ortles 22/4, 20139 Milano, Italy
[4] Dipartimento di Scienze Chimiche, della Vita e della Sostenibilità Ambientale, Università di Parma, 43124, Parma, Italy

* To whom correspondence should be addressed. **Email**: tommaso.bellini@unimi.it ; giuliano.zanchetta@unimi.it ; marco.buscaglia@unimi.it.

Supplementary text

Supplementary figures

Supplementary tables

Supplementary text

S1. Background subtraction in RPI, SPR and protein arrays

Many available techniques to measure specific and nonspecific protein-DNA interactions, including protein microarrays, Surface Plasmon Resonance (SPR) biosensors and the technology RPI used here, rely on the immobilization of DNA strands on a surface. The enormous advantage of localizing the interaction on a surface comes with the problem of how to take into account the possible background signals produced by the surface itself. The largest and least controllable source of spurious signal is the presence of interactions between the proteins and the surface. A good polymer and protein passivation should in principle minimize nonspecific adsorption of proteins on the surfaces of the microarray slide, on the gold SPR sensor surface or on the outer RPI silica layer. However, in all these techniques the signal measured on the surfaces outside of the areas of the spots where DNA is grafted, is generally not negligible for DNA-binding proteins. The proteins from this class typically have a positive net charge, which can induce relevant nonspecific electrostatic interactions with those solid surfaces in water. This raises the question of *whether the nonspecific adsorption on the surface outside the spot has any relevance to the measurements performed in the spots*. Since this topic is of relevance to appreciate the sensitivity to weak binding, we briefly discuss how such nonspecific interactions are typically handled in the signal processing of these detection techniques.

<u>Background treatment in RPI – this study</u>

Our DNA spots are in a regime of rather large surface density, in which the increased concentration of DNA in the spotting buffer does not reflect in a proportional increment of the saturated surface density of Gal4 (Σ_∞), but rather in a decrease of the Gal4-DNA affinity (Suppl. text S5 and Fig. S3), indicating that the various DNA strands screen each other from interactions. In this limit it is hard to imagine how Gal4, which cannot even specifically bind to all DNA probes, could be affected by the sensor surface under the DNA.

This combination of evidences indicates that in our experiments, within the spotted region, the density of dsDNA is sufficient to screen the interactions of Gal4 with the background and that, as a consequence, the surface density of Gal4 binding to DNA is obtained without the need of including the background in the analysis of the signal (see section S3). We also wish to point out that the RPI real-time approach does not require steps of blocking, washing or additional incubation, in each of which the nonspecific interactions with the surfaces could play a role. However, for the sake of comparison with the other techniques, we propose below an alternative analysis in which we also take the background in consideration, against the evidence here reported.

Background treatment in protein microarrays

Measurements of protein-DNA interactions by protein binding microarrays are performed by letting the interaction take place for a given time (of the order of one hour) and measuring the emission of a fluorescently labelled secondary antibody, which is added after blocking and washing. While the qualitative comparison of the binding efficiency to the multitude of distinct DNA strands that this technology enables to test in parallel is straightforward, the conversion of the measured fluorescent intensity on each spot to a binding strength requires a signal processing in which the background has to be considered.

Within the context of zinc-finger proteins, the role of the background in converting measured fluorescence intensity I_F into binding strength has been explicitly described in the works of Bulyk and coworkers. As reported in Ref. (1), Fig 4D and in Ref. (2) Fig. 2b, such quantitative conversion is accurate for strong binding, where variations in K_D cause big changes in I_F, while it is quite imprecise for large K_D, where the hyperbolic I_F vs. K_D dependence has a small derivative. The quantitative conversion in this range is made even more imprecise by the presence of a background fluorescence due to nonspecific binding to the surface of the DNA-binding protein or of the tagged antibody, to be subtracted. As apparent from the figures referred above, the determination of the conversion curve in this range is very imprecise.

Background treatment in SPR imaging

Despite the optical mechanism being different (thin layer reflectivity vs. absorption by surface plasmons), both RPI and SPR imaging experiments measure the intensity reflected by the sensor surface, on which receptors (in this case dsDNA) are immobilized on spots. Thus, topics related to signal treatment and background corrections in SPR imaging could be analogous to our experiment. However, the choices made in SPR imaging are typically different than ours. This is illustrated in Ref. (3), in which the interactions of Gal4 with a panel of dsDNA immobilized on spots are measured. In this work the authors perform a straight subtraction of the whole background reflectivity curve to the reflectivity curves measured in all spots. We however cannot see how this could justify assuming that the Gal4 interaction with control dsDNA is simply summed up to the interactions with the underlaying polymer substrate. In the specifics of Ref. (3) this choice is particularly questionable since, as apparent in their Fig. 5, the amplitude and kinetics of the interactions of Gal4 to the nonspecific dsDNA and to the polymer are basically identical. This would amount to assuming that Gal4 interacts with the sensor surface through a layer of generic dsDNA with which it does not interact at all, which is of course unrealistic.

Overall, not only we believe that the issue of background subtraction is much better handled in RPI – where the bare surface reflectivity is incorporated straight away into the data treatment, but we would also like to exploit this SPR imaging-to-RPI comparison to point out that any poorly

justified background subtraction affects much more the determination of the weak nonspecific interactions than the characterization of specific interactions. This is a crucial factor in evaluating the performance of the various methods to measure the DNA interactions of transcription factors (TF).

<u>Background treatment in RPI – an alternative approach</u>

As specified above, various observations support the notion that the molecular adsorption on the polymer coating outside the spotted areas should not be taken into account when extracting from the RPI signal (local reflectivity) the amount of Gal4 interacting with the spotted DNA of our signal. However, for the sake of comparison to the signal processing performed in the other techniques, we have evaluated how much subtracting the background would affect the values of equilibrium and kinetic binding coefficients. We performed the analysis of the Gal4 binding curves at ionic strength of 150 mM after subtracting the signal obtained from the background area surrounding each spot. We adopted two different approaches: (i) we subtracted the raw reflectivity signal, similarly to Ref. (3); (ii) we subtracted the surface density signal (see section S3). The comparison of the resulting surface densities is reported in Fig. S4. In both cases, we obtained similar or lower values of K_d, within a factor of two, and faster kinetics, up to a factor of ten, relative to the case of no background subtraction. The extracted value of K_2, given by the ratio of the K_d measured for the specific and nonspecific spots and indicating the depth of the inner well in the NW model, also increases within a factor of two relative to the case of no background subtraction. Overall, we conclude that the adoption of a particular background subtraction strategy in the analysis of the binding curves can provide a limited but well detectable effect on the equilibrium parameters and a stronger effect on the kinetic parameters. Therefore, in general, background correction strategies applied to protein-DNA binding data should be evaluated with extreme caution. As explained above, our dense layers of DNA probes are expected to effectively hide the underlying sensor surface, hence we ascribe the signal from the spot of nonspecific DNA sequence entirely to the nonspecific protein-DNA interaction, without subtracting the signal measured outside the DNA region.

S2. Binding of GAL4 at different temperatures

In order to estimate the temperature dependence of the specific binding of GAL4, we performed experiments analogous to that reported in Figure 2 at different temperatures T. Figure S8 shows that at 50 nM GAL4 the amplitude on nonspecific spots is rather constant from 15°C to 45°C, whereas the amplitude on spots hosting the specific strands strongly decreases and the binding curves become progressively similar to that measured on nonspecific spots. From the analysis of the concentration dependence of the equilibrium amplitudes we obtained the ratio between the specific and nonspecific equilibrium constants, from which we compute $K_2(T)$, reported in Figure

4. The behavior of K_2 confirms a strong temperature dependence of the specific binding of GAL4 to the consensus sequence.

S3. RPI measurements

The RPI measurements were performed by using the experimental set-up and the analysis procedure described in ref. (4). Briefly, Gal4, previously equilibrated in buffer, was injected into the RPI cartridge to reach a final concentration *c* from 0.08 nM up to 50 nM. We avoided larger protein concentrations that can result in aggregation. All the experiments were performed at 30 °C under stirring, except those meant to test for temperature dependence (in Section S2). The reflected light images of the spotted RPI sensing surface were analyzed by a custom Matlab program to obtain the brightness $u_s(t)$ of the spot regions as a function of time *t* and convert it into the total mass surface density of molecules $\sigma(t)$. The surface density of the proteins bound to the immobilized DNA strands was obtained as $\sigma_p(t) = \sigma(t) - \sigma_{DNA}(t)$, where $\sigma_{DNA}(t)$ is the surface density of oligonucleotides measured before the addition of Gal4 in solution.

The conversion of the brightness of the RPI image pixels into surface density is performed according to:

$$\sigma(t) = \sigma^* \sqrt{\frac{u_s(t)}{u_0} - 1} - \delta\sigma \tag{S1}$$

where σ^*, u_0 and $\delta\sigma$ are obtained according to ref. (4) from the physical parameters of the RPI sensor, the refractive index of the solution, and the density and refractive index of a compact layer of biomolecules on the surface (5)(6).

Figure S1 reports the surface density at equilibrium (panels A-C) and the initial slope (panels D-F) of three separate experiments repeated in the same conditions (30°C and 150 mM NaCl). The average values and the standard deviations of K_d and k_{off} obtained from the fits are reported in Figure 2D.

S4. Binding of GAL4 to different control sequences

We explored the interactions of GAL4 with DNA double helices that contain its consensus sequence 5' CGG AGG ACA GTC CTC CG 3', chosen based on previous studies. In particular we used the same sequence as the crystallographic model taken as reference for molecular dynamics simulations (7) and compared it with the behaviour observed with sequences of the same length, in which only the consensus tract has been changed. We also tested additional controls: a single stranded sequence and another double strand hairpin with a different sequence along the entire strand. All DNA sequences used in this work are reported in Table S1. We measured the time evolution of the amount $\sigma_p(t)$ of molecular mass of protein accumulating on

the specific spot GAL4-HP and on three different nonspecific spots, NSP-HP, CTRL-SS and CTRL-HP, following the injection of GAL4 in the measuring cell . The result is shown in Figure S2 and confirms that the specific spots capture a much larger amount of protein and the three different control sequences provide a similar response to the addition of GAL4 in solution. The slightly higher σ values observed for NSP-HP compared to CTRL-HP at the highest Gal4 concentration (50 nM) are likely due to the presence in the NSP sequence of a cryptic site (CGG starting at position 44). Such a motif, as well as the consensus motif, could be detected by scanning of GAL4, NSP and CTRL sequences with the CIS-BP sequence scanning tool based on position weight matrix for TF binding (http://cisbp.ccbr.utoronto.ca/). Specifically, we obtain a CIS-BP score of 19 for the GAL4 sequence – well above the conventional value threshold of 8 to gauge TF binding sites, a score of 6.1 for the NSP sequence and a score below 4 for the CTRL sequence. Specifically, we find the dissociation coefficient of CTRL-HP to be about 30% larger than NSP-HP, a difference of the order of the intrinsic uncertainty on these figures. Considering the relatively high frequency of occurrence of a CGG motif in random sequences, we decided to use for more in-depth analysis the NSP sequence, as more representative of the selectivity challenge that transcription factors normally face. Although GAL4 should not bind the CTRL-SS sequence, as it is an ssDNA, we see an increase in the σ signal over time. These values, observed for CTRL-SS, are probably due to the electrostatic interaction. This also suggests that the nonspecific interaction on the NSP-HP and CTRL-HP sequences is mostly due to electrostatic interaction. This is evidenced by the fact that the NSP-HP sequence shows a Gal4's binding curves similar to the CTRL-SS one.

S5. Effect of surface density of probes

Surfaces were prepared with different concentrations of DNA in the spotting buffer. The resulting surface probe densities displayed a small increase with the spotting concentration of DNA above 5 μM. The analysis of the binding curves enabled determining the values of the saturated surface density of Gal4 (Σ_∞) and the dissociation constant K_d. The measured Σ_∞ is proportional to the surface density of DNA probes. As reported in Figure S3A, we observed a small increase of K_d (smaller binding strength) with the increase of probe potting concentration (or probe surface density), indicating that the increased surface crowding slightly hinders the interactions. As shown in figure S3B, at 10 nM protein, the fraction of probes bound by GAL4 is on average 0.08 for the nonspecific sequence and 0.22 for the specific sequence. At asymptotically large protein concentration, the fraction of GAL4-bound probes averaged around 1 for both the specific and the nonspecific strands with small variations dependent on probe concentration.

S6. Complete solution of the Nested-Well model

The Nested-Well (NW) model embodies a concept - often proposed in the context of transcription factors - that binding to the cognate site takes place through nonspecific interactions. Accordingly, the model describes the onset of specific interactions as a two-step process: first a looser binding takes place, corresponding to the interaction of the protein with a generic DNA double strand, followed by a tighter binding in a restricted conformational space, in agreement with the notion that specific binding involves a well-defined mutual positioning of protein and DNA and the adoption of specific conformations. Therefore, the protein-DNA system can be described by a three-state model:

$$unbound \leftrightarrow nonspecific\ binding \leftrightarrow specific\ binding \tag{S2}$$

The overall kinetics of the NW model is provided by change over time of the specific and nonspecific populations according to:

$$\begin{cases} \dfrac{d\sigma_1}{dt} = k_{on1}c(\Sigma_\infty - \sigma) - k_{on2}\sigma_1 + k_{off2}\sigma_2 - k_{off1}\sigma_1 \\ \dfrac{d\sigma_2}{dt} = k_{on2}\sigma_1 - k_{off2}\sigma_2 \end{cases} \tag{S3}$$

where the kinetic constants are as defined in the main text and $\sigma = \sigma_1 + \sigma_2$.

The resulting surface density measured by RPI, which detects at the same time specifically and non-specifically bound proteins, is given by

$$\sigma(c,t) = \Sigma(c)\left(1 - B\,e^{-t/\tau_L} - (1-B)\,e^{-t/\tau_S}\right) \tag{S4}$$

where

$$\tau_L^{-1} = \tfrac{1}{2}\left(\lambda - \sqrt{\lambda^2 - 4Q}\right)$$
$$\tau_S^{-1} = \tfrac{1}{2}\left(\lambda + \sqrt{\lambda^2 - 4Q}\right)$$
$$\lambda = k_{off,1} + k_{off,2} + k_{on,2} + k_{on,1}c$$
$$Q = k_{off,1}k_{off,2} + k_{on,1}k_{on,2}c + k_{off,2}k_{on,1}c \tag{S5}$$

and

$$B = \frac{1 - \tau_L k_{off,2} - \tau_L k_{on,2}}{(\tau_L - \tau_S)(k_{off,2} + k_{on,2})} \tag{S6}$$

It is worth noticing that in the limit $k_{on,2} \to 0$, i.e. in the absence of specific binding, $B \to 0$, so that the response is a single exponential with characteristic time $\tau_S^{-1} \to k_{off,1} + k_{on,1}c$, as expected for the signal growth in the absence of the specific well. Fig. S6 shows the ratios τ_S/τ_1 and τ_L/τ_1, where $\tau_1 = (k_{on1}c + k_{off1})^{-1}$, as a function of the depth of the internal well K_2 and of its escape rate k_{off2}, with a given choice of K_1 and k_{off1}. As visible in the figure, $\tau_S < \tau_1$ and $\tau_L > \tau_1$ always. Fig. S6 also shows the behaviour of B (magenta lines, right y axis). In the limit of small K_2, all proteins enter the internal well, $B \to 1$ and the kinetics becomes instead dominated by the long decay time, that grows progressively large (panel A). At moderate K_2, τ_L (the dominating response time)

has a partial dependence on k_{off2}: when k_{off2} is small, τ_L becomes large as expected because of the slower escape time from the inner well; when k_{off2} is large, τ_L reaches a limiting value $\tau_{L\infty}>\tau_1$. $\tau_{L\infty}$ depends on K_1, K_2 and τ_1, and corresponds to the time involved in the escape from the outer well of the non-specifically bound proteins, that in this limit are always in equilibrium with the specifically bound ones.

The kinetics predicted by the NW model can be compared with observations. Data shown in Fig. 1 of the main text were simultaneously fit to the model (continuous lines). In the procedure, we held Σ_∞ fixed to the value determined from the analysis of the Langmuir isotherms (Fig. 2B), and used K_1, K_2, k_{off1} and k_{off2} as fitting parameters. We find a good agreement with the data, indicating that the NW model captures the differences in both binding strength and kinetics. K_1 and k_{off1} obtained by the best fit are compatible with those in Fig. 2, while K_2 agrees with the data in Fig.3D. The result obtained for k_{off2} requires some further discussion.

In the fit process, we find that the amplitude $B \approx 1$, a limit obtained when k_{off2} is large enough. In this condition, the kinetics is given by τ_L (with $\tau_L > \tau_1$) and becomes independent from k_{off2}, while still depending on K_2 as

$$\tau_{L\infty} \equiv \tau_L(k_{off2} \to \infty) = \tau_1\left(1 + \frac{K_1}{K_1K_2+c+K_2c}\right) \tag{S7}$$

$\tau_{L\infty}$ is the fastest kinetics that can be obtained with any value of k_{off2} at fixed K_1 and K_2. The existence of such a limiting value can be understood as follows. At any given moment, only a fraction $(1-K_2)$ of the proteins are in the outer well, i.e. non-specifically bound. Since the unbinding process occurs through the outer well only, the escape rate is reduced by that same $(1-K_2)$ amount. This results in a slower process to equilibrium. This limiting kinetics can be expressed via the quantities $k_{on\infty}$ and $k_{off\infty}$, to be compared to the measured k_{on} and k_{off}. By taking the limit to $c \to 0$ of $\tau_{L\infty}^{-1}$ we obtain

$$k_{off\infty} \approx k_{off1}\left(\frac{K_2}{K_2+1}\right) \tag{S8}$$

In practice, in the fitting process we find that k_{off2} is within the limit of large values, and therefore we cannot determine accurately its specific value. This difficulty can be appreciated in Fig. 1B of the main text, where the dashed lines represent the signal expected for $k_{off2} \to \infty$. As apparent in the figure, we find the limiting value to be so close to the data to prevent any reliable assessment of k_{off2}. As a reference, the limiting behaviour at large k_{off2} is obtained, with deviations of less than 10%, (i.e. $k_{on} \geq 0.9 \times k_{on1}$) when $k_{off2} > k_{off1}$. This sets a limit for k_{off2}, which corresponds to the notion that the residence time of Gal4 on its consensus sequence is shorter than $k_{off1}^{-1} \approx 300$ *s.*

At the same time, the rate k_{on} measured for the NW model becomes $k_{on} = k_{on\infty} = k_{on1}$ at the limit of large $k_{off,2}$. This is shown in Fig. S7, where we plot the ratio k_{on}/k_{on1} as a function of the

ratio $k_{off,2}/k_{off,1}$. The curve in the figure does not depend on K_1 while it depends on K_2 very mildly.

S7. All-atom molecular dynamics simulations

Simulation protocols and analysis

To model Gal4 transcription factor protein, we started with the crystallographic structure of the DNA-bound protein (PDB ID: 3COQ). For DNA sequences, the free structure was energy optimized using the internal/helicoidal variable modelling JUMNA (8) with the AMBER par98 force field with the BSC0 modifications (9) and a Generalized Born continuum solvent model using the parametrization of Tsui and Case (10), which comprises added salt effects via a Debye-Hückel term. JUMNA was used to construct complexes within oligomers containing the experimentally studied binding sequences, maintaining the conformation of the protein and of the protein-dsDNA interface.

Molecular dynamics simulations were performed with the GROMACS 5 package (11) using the Amber 99SB-ILDN force field for proteins (12) and with the BSC0 modifications for the nucleic acids (9). We performed microsecond long, all-atom simulations on GAL4 alone, the specific DNA sequence alone, the nonspecific DNA sequence alone, GAL4 in complex with the specific DNA sequence (specific complex) and GAL4 in complex with the nonspecific DNA sequence (nonspecific complex). The single proteins, nucleic acids and the complexes were placed in a cubic box and solvated with TIP4P water molecules (13) to a depth of at least 15 Å. The solute was neutralized with potassium cations and then K^+Cl^- ion pairs (14) were added to reach a physiological salt concentration of 0.15 M. Zn^{2+} ions were modelled with the cationic dummy atoms as developed by Pang (15). Long-range electrostatic interactions were treated using the particle mesh Ewald method with a real-space cut-off of 10 Å. The HB lengths were restrained using P-LINCS, allowing a time step of 2 fs. The translational movement of the solute was removed every 1000 steps to avoid any kinetic energy build-up. After the energy minimization of the solvent and the equilibration of the solvated system for 10 ns using a Berendsen thermostat (τ_T = 1 ps) and Berendsen pressure coupling (τ_P = 1 ps) (16), the simulations were carried out in an NTP ensemble at a temperature of 310 K and a pressure of 1 bar using a Bussi velocity-rescaling thermostat (17) (τ_T = 1 ps) and a Parrinello-Rahman barostat (18) (τ_P = 1 ps). During minimization and heating, the protein backbone and DNA heavy atoms were kept fixed using positional restraints. These restraints were slowly relaxed during the equilibration. The length of the simulations was of 1000 ns. For the specific and nonspecific complexes, we performed two repeats.

The conformational analysis of dsDNA was performed using Curves+ (19), which provides a full set of helical, backbone and groove geometry parameters. Parameters are grouped into five sets: (i) intra-base pair (shear, stretch, stagger, buckle, propeller, opening); (ii) BP-axis (Xdisp, Ydisp, inclination and tip); (iii) inter-BP (shift, slide, rise, tilt, roll, twist); (iv) backbone (in the 5′→3′ direction for each nucleotide, α P-O5′, β O5′-C5′, γ C5′-C4′, δ C4′-C3′, ϵ C3′-O3′, ζ O3′-P, the glycosidic angle χ C1′-N1/N9 and the sugar pucker phase and amplitude); (v) groove (minor and major groove widths and depths). All Curves+ parameters are output in a file containing a single record for each snapshot in each oligomer, which allowed us to perform further statistical analysis using Canal (19).

At each frame of the trajectory we identified the HB between the protein and the DNA sequence based on cut-off for the Donor-H...Acceptor distance and angle according to the Wernet-Nilsson "cone" criterion as implemented in mdtraj (distance between donor and acceptor heavy atoms below a given distance cut-off, dependent on the angle made by the hydrogen atom, donor, and acceptor atoms) (20). Then we computed the average number of HB, their occupancy and fluctuations.

Movement of the protein along DNA sequences

The movements of the protein along the nonspecific DNA sequence are confirmed by the increase of the RMSD for the nonspecific sequence in Figure 5B. In order to better characterize these movements, we aligned the DNA sequences along their z-axis, and we computed the displacement along the z-axis of a given amino-acid and the center of mass of a pair of bases. We chose the amino-acids and the base pair involved in hydrogen bonds. Figure S9 shows the displacement along the z-axis of the LYS18.A with respect to the base pair G10:C59 and the LYS18.B with respect to the base pair G10:C59. The displacement along the z-axis of the LYS18.A with respect to the base pair G10:C59 in the case of GAL4 in complex with the nonspecific DNA sequence allowed us to determine the change of binding sites. Based on the LYS18.B with respect to the base pair G10:C59, we compared the stability of the contacts between GAL4 and the specific and nonspecific DNA sequences. In the former the protein is more stable when bound to the DNA. On the contrary, in the latter, the protein moves around an average position hence the contacts are less stable.

DNA properties

In the case of the specific DNA sequence, we observed a large change in the average bending of the DNA toward the protein for one repeat (56.5° versus 41.3° in the isolated DNA oligomer) and only a slight change in the other one (43.2°). This is also observed in other inter-base-pair quantities (like twist) and in the groove dimensions as shown in Figure S10. On the contrary, in the case of the nonspecific DNA sequence, we only observed a negligible bending of the DNA

toward the protein (34.8° and 34.0° versus 33.6° in the isolated DNA oligomer). The inter-base-pair parameters were also mostly unaffected by the presence of the protein as well as the groove dimensions as shown in Figure S11.

Solvation energy

To characterize the protein-DNA interface and estimate the solvation contribution to thermodynamics, we also determined the number and distribution of water molecules within 4 Å from both the protein and the DNA sequences. We computed the number of water molecules lost for the formation of the complex, Δn_{water}, as follows:

$$\Delta n_{water} = < n_{w,prot+DNA} > -(< n_{w,prot} > + < n_{w,DNA} >) \quad \text{(S9)}$$

where $< n_{w,prot+DNA} >$, $< n_{w,prot} >$ and $< n_{w,DNA} >$ are the average numbers of water molecules within a distance of 4 Å from the protein-DNA complex, the protein alone and the DNA sequence respectively.

Interfacial water molecules, which easily exchange with water molecules from the bulk solution along the MD simulation, compete for the formation of specific HB. The number of such interfacial water molecules is 147±11 and 153±14 for the specific and nonspecific complex, respectively. Figure S12 shows the water molecules computed for some representative structures. The change of the binding site also influences the distribution of water molecules between the protein and the DNA molecules. The number of molecules lost in the formation of the specific and nonspecific complexes (Δn_{water}, Eq. S9) is equal to -182 and -157. This result suggests that the number of HB between water-DNA and water-protein is reduced more in the formation of the specific complex than in the nonspecific one.

The solvation free energy can be defined as the amount of energy associated with dissolving a solute in a solvent as per the following:

$$\Delta G_{solvation} = \Delta G_{ps} + \Delta G_{np} \quad \text{(S10)}$$

where ΔG_{ps} is the polar solvation energy, which can be estimated by solving the Poisson-Boltzmann equation. The second term, ΔG_{np}, is the non-polar solvation energy component and is usually calculated by means of a linear relationship between the former and the SASA (Solvent Accessible Surface Area) of the single protein or DNA and protein-DNA complexes:

$$\Delta G_{np} = a\text{SASA} + b \quad \text{(S11)}$$

where *a* and *b* are two energetic parameters, commonly assumed to be a = 0.00542 kcal mol^{-1} $Å^{-2}$ and b = 0.920 kcal mol^{-1} (21).

Electrostatic calculations via Poisson-Boltzmann equation model

The Poisson-Boltzmann (PB) equation model treats the solvent as a continuum medium with high dielectric constant. Biomolecules are considered as cavities with low dielectric constant made of

charged atoms. Ions in the water phase are modeled as non-interacting point charges and their distribution obeys the Boltzmann law. The resolution of the PB equation allows us to determine the electrostatic potential around the proteins under investigation (22, 23), the electrostatic (ΔE_{el}) and polar solvation energy (ΔG_{ps}).

In our calculation, the Poisson-Boltzmann equation was solved using DelPhi 8.0 (24). The biomolecule is assumed to be a homogeneous medium with low dielectric constant ε_{in} = 4 whereas the solvent is modeled by a high dielectric constant (ε_{out} = 80 at 298°K for water). A monovalent salt of concentration of 0.15 M has been taken into account. We assigned the charges using the same force field exploited in MD simulations.

Characterization of the interfaces

To characterize the protein-DNA interfaces, we computed the average number of hydrogen bonds in the starting structures and along the trajectory at the interface between the DNA sequences. Tables S2 and S3 summarize the HB detected in our MD simulations. In the presence of the specific DNA sequence, the average number of active HB is only slightly higher ($\langle n_{HB,s(1)} \rangle$= 21 ± 3, $\langle n_{HB,s(2)} \rangle$= 18± 3, $\langle n_{HB,ns(1)} \rangle$= 15 ± 3, $\langle n_{HB,ns(2)} \rangle$= 16 ± 3). As regards the number of HB with an occupancy 30% or more, the figure is instead much larger for the specific DNA sequence ($n_{HB,s(1)}$ = 16, $n_{HB,s(2)}$ = 11, $n_{HB,ns(1)}$ = 3, $n_{HB,ns(2)}$ = 8). The original contacts are mostly preserved in the specific DNA sequence, while they are almost completely lost in the nonspecific one.

Protein secondary structure

To better characterize the protein and its conformational changes upon binding, we computed the secondary structure along the MD trajectories for each residue at each snapshot (25). We classified the secondary structures in 3 different categories: helical, sheet and coil. Helical secondary structure includes α-helix, 3_{10} helix and π helix. For sheet secondary structure we refer to hydrogen bonded turn and extended strand in parallel and/or anti-parallel β-sheet. For each residue, we then computed the percentage average propensity (SS propensity %) to allow the comparison with the protein starting structure and between the different trajectories. We also computed the difference between the secondary structural propensity (ΔSS propensity %) between the SS propensity of the protein in complex with the DNA sequence and the protein alone. The results are reported in Fig. 5A.

Entropic terms

We considered explicitly only the entropy related to the biomolecules, in particular the configurational (or solute) entropy although other entropy contributions exist. This approach has

been widely used in other studies (26, 27). Another contribution to the entropy is given by the rotational-translational entropy loss. The magnitude of rotational-translational entropy loss (ΔS_{RT}) is still a subject of debate with estimations ranging from 0 (28) to ~50 cal/mol/K (15 kcal/mol at 300 K) (29). It mainly depends on the number of interacting components (29, 30). In the complexes we treat, the dissociated state always involves a double-stranded DNA and a protein monomer or dimer. In all the cases studied, protein dimerization occurs before interaction with DNA. Therefore, this term can be considered constant for the set of complexes and has not been treated here.

The configurational entropy mostly takes into account the side-chain entropy and the folding entropy. The solute entropy was estimated from the covariance matrix of the atomic-positional fluctuations and the Schlitter formula (31) as implemented in GROMACS5 after superimposition of all the frames on the starting one. The calculation of entropy for different regions of GAL4 is a very delicate point. Generally, an arbitrary set of atom coordinates can be used in the calculation of the covariance matrix. However, when using subsets of the degrees of freedom of a system in the entropy calculation, one has to be aware that the entropy is, in principle, a nonadditive quantity. By decomposing the total entropy into parts, one neglects the correlation between the parts. In this case, for the calculation of the entropy per residue, a separate covariance matrix for each residue had to be used. Any correlation between the residues is, therefore, ignored and the entropies per residue do not add up to the configurational entropy of the entire protein. The sum of the configurational entropies of all residues is larger than the total configurational entropy of the protein, because the correlations that lower entropy are neglected. A similar effect can be observed if we separate the entropy of the protein and that of the DNA sequence.

The change in conformational entropy can be related to different factors, among them the folding, backbone and side-chains conformations in the protein. First, we decompose the total entropy of the complexes into two parts: the entropy of DNA ($S_{DNA,complex}$) and the protein ($S_{protein,complex}$) alone. We also computed the correlation between the protein and DNA. This can be obtained by taking the difference between the sum of the protein and DNA entropies and the configurational entropy of the complex ($S_{complex}$). While $S_{complex}$ contains all correlations between the DNA and protein atoms, the values calculated using only either subsets do not. The difference therefore gives the decrease in entropy due to these correlations:

$$S_{protein/DNA,complex} = S_{DNA,complex} + S_{protein,complex} - S_{complex}. \quad \text{(S12)}$$

We also computed the change in entropy of DNA ($\Delta S_{DNA} = S_{DNA,complex} - S_{DNA,unbound}$) and protein ($\Delta S_{protein} = \Delta S_{protein,complex} - S_{protein,unbound}$) upon binding separately and their sum ($\Delta S_{complex,protein/DNA} = \Delta S_{DNA} + \Delta S_{protein}$). Table S4 summarizes these results. From this analysis, we observed that the correlation between DNA and the protein in the conformational entropy represents a small fraction of the total conformational entropy of the complex and the values obtained for the specific and nonspecific complex are comparable. Hence, we can compare the change of entropy of DNA

and the protein upon binding. The positive entropy for the nonspecific complex seems to be due to the change of conformational entropy of the protein which is much larger than the loss of entropy of DNA. On the contrary, for the specific sequence, although the loss of entropy of DNA is in absolute value slightly lower than the increase of the entropy of the protein upon binding, the cooperative effects may explain the negative change of entropy upon binding.

Several factors may contribute to the positive change of configurational entropy of the protein, like the penalty in sidechain entropy upon binding for the decrease in surface accessible area, but also the penalty/gain in backbone and sidechain entropy in the case of folding/unfolding of a part of the protein upon binding. The configurational entropy due to sidechains is known to be proportional to the solvent-accessible area of the residue and in this case, we predict a loss of side-chain conformational entropy occurring upon binding at the DNA-protein interface.

To get insights on the effect of the folding, we computed the change in the secondary structure propensity as explained above. By comparing the secondary structure propensity in the protein alone and in the complex, we can observe a similar behaviour for the specific and nonspecific complex. In particular, we can identify two regions, namely the Zn part (8-36) and the unfolded linker (36-50). In the former, the secondary structure in the complex is similar to the one in the crystallographic structure On the contrary, in the protein alone the helical region is not well conserved, and the unfolding region assumed a turn secondary structure, so we can predict a gain in entropy for both complexes. For the latter, the region is unfolded in both complexes, while it assumes a turn secondary structure when it is alone. Hence, we can conclude that the unfolding in the linker has a major role in increasing the entropy for both systems.

Energy characterization

We performed two repeats for each complex. Hence, we computed for each energetic contribution the average and the standard deviation. We also verified that the convergence was reached by applying a block averaging for the energetic terms on the single trajectory using the approach implemented in GROMACS (32). Table S5 summarizes some of the energetic contributions to binding energy estimated from the all-atom molecular dynamic simulations detailed above: the non-polar contribution to the solvation energy (ΔG_{np}), the van der Waals energy contribution (ΔE_{vdw}) modelled using a Lennard-Jones potential function (33), the electrostatic energy modelled using a Coulomb potential function (ΔE_{el}) (33), the polar solvation energy (ΔG_{ps}) and the solute entropy. For sake of clarity, we combined in one term the electrostatic energy and the polar solvation energy and in another term the van der Waals energy contribution and the non-polar contribution to the solvation energy (ΔG_{NP}). The solute entropy stabilizes the nonspecific complex in both repeats. In contrast, the entropic term is highly destabilizing for the specific complex. All the complexes are similarly stabilized by the non-polar contribution to the solvation energy (ΔG_{np}). Moreover, both complexes are stabilized by the van

der Waals energy contribution (ΔE_{vdw}) but in the specific complex the energy is larger the nonspecific one.

S8. Materials

Full Gal4 protein has a length of 881 amino acids and comprises a Zn–Cys binuclear cluster type DNA-binding domain (zinc finger), a linker domain, a dimerization domain and two acidic activation domains. We study a smaller and stable recombinant *S. cerevisiae* GAL4 N-terminal fragment, comprising amino acids 1-147, purchased from Abcam (Abcam, Cambridge, UK). Gal4(1-147) specifically binds DNA as a dimer. Oligonucleotides were purchased from Integrated DNA Technologies (IDT) (Integrated DNA Technologies, Coralville, IA, USA) with Ultramer synthesis. All buffers and reagents were purchased from Sigma-Aldrich (Sigma-Aldrich, St. Louis, MO, USA) and prepared according to common protocols using Milli-Q pure water.

Amine-terminated oligonucleotides were suspended in spotting buffer (Na_2HPO_4 pH 8.5 150 mM) at concentrations of 5 µM, 10 µM and 30 µM and deposited on the RPI sensor surface, coated with a multifunctional copolymer, by an automated noncontact dispensing system (sciFLEXARRAYER S3, Scienion AG, Berlin, Germany) (4). After overnight incubation, the chip surface was rinsed with blocking buffer (Tris HCl pH 8 10 mM, NaCl 150 mM, ethylenediamine 50 mM) and distilled water and then dried. The RPI sensor cartridges were prepared by gluing the spotted chips on the inner wall of 1-cm plastic cuvettes. Gal4 was suspended before use in measuring buffer (Tris HCl pH 7.5 50 mM, Tween 20 0.02%, NaN_3 0.02%, $ZnSO_4$ 200 µM, NaCl from 50 to 250 mM).

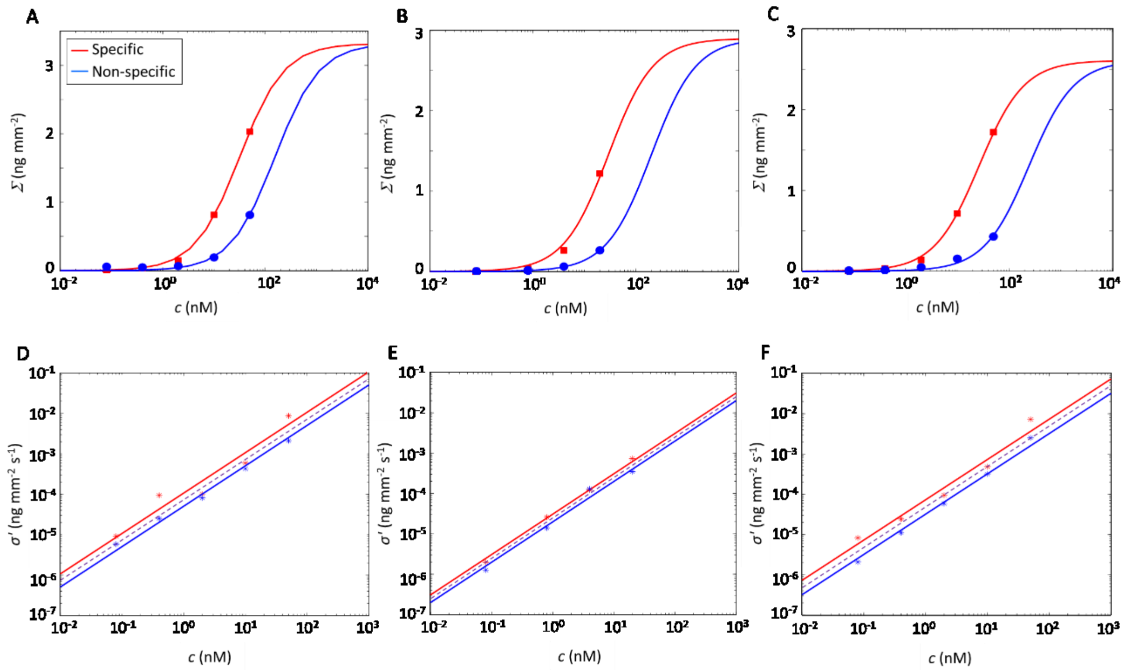

Figure S1. Analysis of repeated experiments on Gal4-DNA interaction. The plots report three separate experiments used to calculate the average and standard deviation of K_d and k_{off} shown in Figure 2D. (A-C) Amplitude $\Sigma(c)$ for specific (red) and nonspecific probes (blue) upon increase of protein concentration. Lines are fits with the Langmuir model of Eq. 2. (D-F) Initial slope $\sigma'(c)$ of the same experiments of panels A-C for specific (red) and nonspecific (blue) probes. Continuous lines are linear fits to the data with the same color, and dashed lines are global linear fits to both specific and nonspecific data, as discussed in the text.

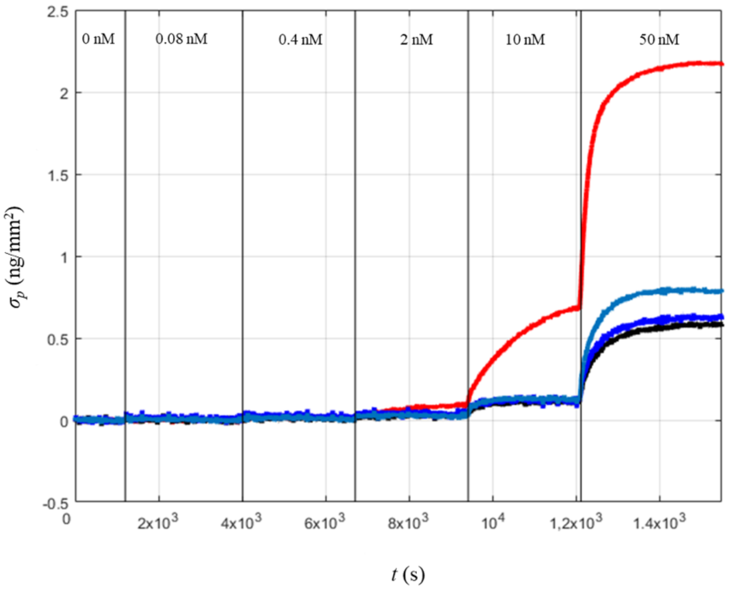

Figure S2. RPI analysis of GAL4-DNA interaction. Increase of the surface density measured over time upon protein binding to specific sequence GAL4-HP (red), and nonspecific sequences NSP-HP (light blue), CTRL-SS (black) and CTRL-HP (dark-blue), for increasing concentration of GAL4 in solution (ionic strength I_s = 150 mM, T = 30 °C, and spotting concentration of DNA 10 μM). At the time indicated by the vertical lines, GAL4 concentration was increased step-wise from 0 to 50 nM, as indicated. The analysis of these data by the Langmuir model of Eq. 2 yielded the following equilibrium dissociation constants: K_d ≈ 31 nM for GAL4-HP, K_d ≈ 161 nM for NSP-HP, K_d ≈ 208 nM for CTRL-HP, K_d ≈ 212 nM for CTRL-SS.

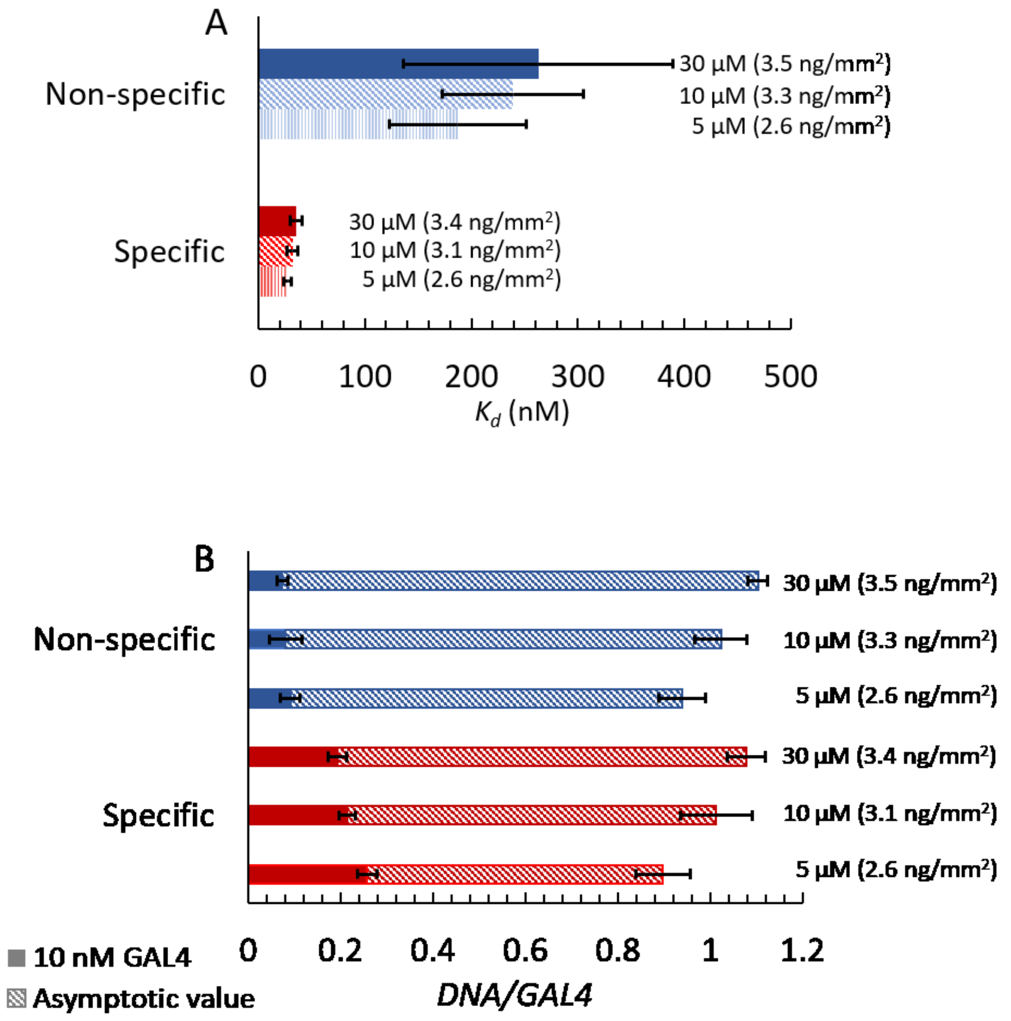

Figure S3. Effect of probe surface density on the equilibrium constant for dissociation and on DNA probe fraction bound by GAL4. Bar graph showing (A) the dissociation constant K_d for GAL4-DNA binding and (B) DNA probe fraction bound by GAL4 at 10 nM (solid filling) and at asymptotically large concentration (patterned filling) on specific and nonspecific DNA duplexes immobilized with different spotting concentrations. The corresponding probe surface density is reported between parentheses. The error bars represent SD of the average of three values from separate experiments.

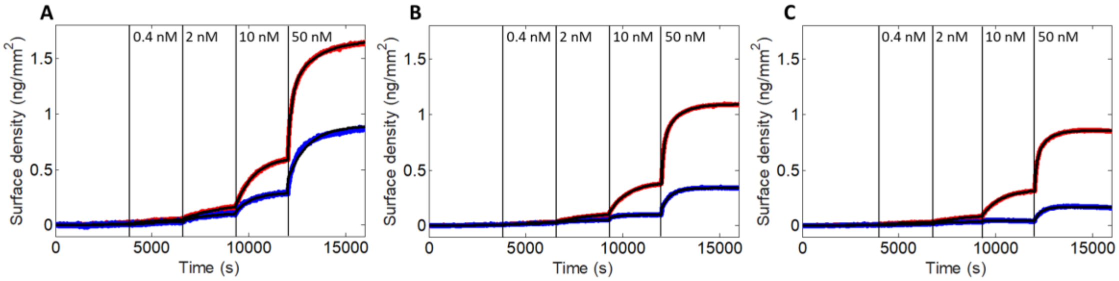

Figure S4. Effect of background subtraction on GAL4 binding curves. Surface density of GAL4 measured on spots of specific (red) and nonspecific (blue) DNA sequence, obtained (A) without background subtraction or subtracting (B) the reflectivity or (C) the surface density of the area surrounding the spot to the reflectivity or surface density of each spot, respectively. The binding curves were measured upon increasing concentration of GAL4 in solution as indicated in the figures, at ionic strength I_s = 150 mM, T = 30 °C, and spotting concentration of DNA 10 μM. Black lines are fit of the experimental curves with Eq. 1.

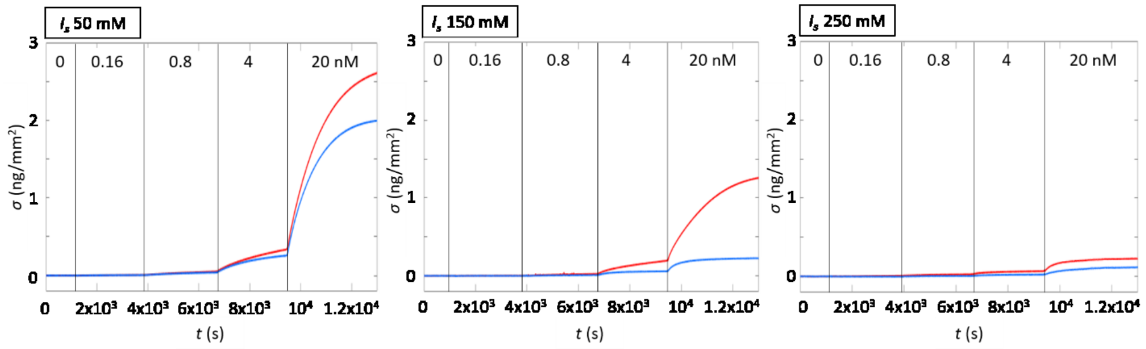

Figure S5. Gal4-DNA binding curves at different ionic strengths. Increase of the surface density upon protein binding to specific (red) and nonspecific (blue) hairpin sequences for increasing concentration of Gal4 in solution at different ionic strengths Is: 50 (left), 150 (center) and 250 mM NaCl (right).

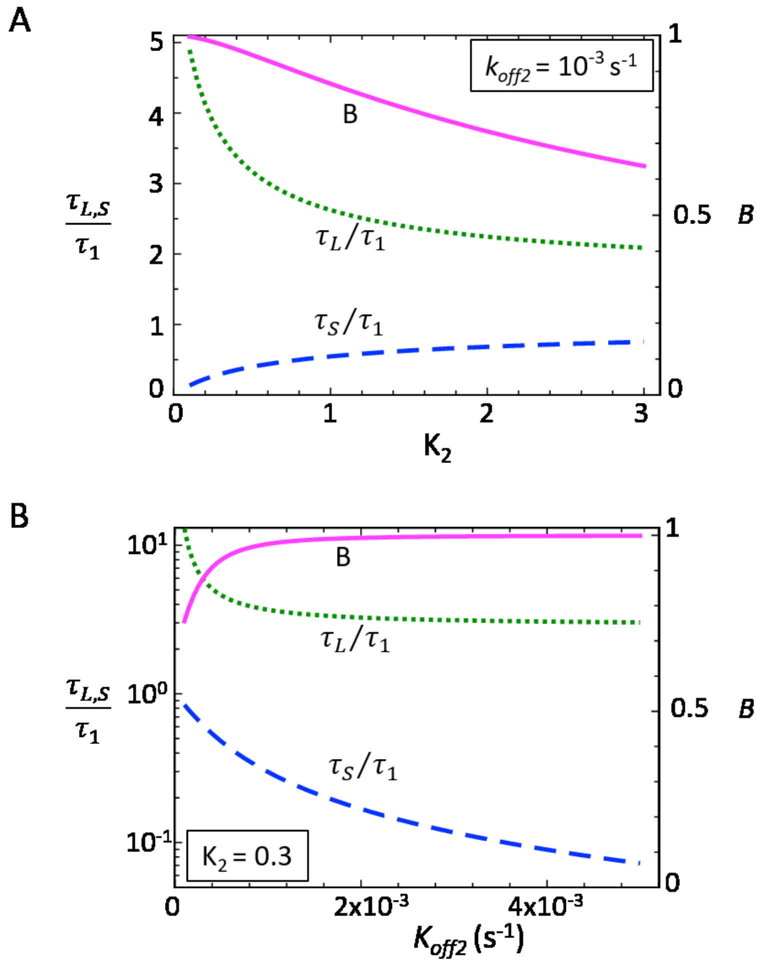

Figure S6. Dependence of the two characteristic times τ_L and τ_S relative to the nonspecific characteristic time τ_1, on the specific/nonspecific equilibrium binding constant K_2, for k_{off2} = 10^{-3} s^{-1} (A) and on the rate k_{off2} of unbinding from the consensus sequence for K_2 = 0.3 (B). In both cases k_{off1} = 10^{-3} s^{-1} and K_1 = 100 nM. Always $\tau_L / \tau_1 > 1$ and $\tau_S / \tau_1 < 1$. Right axes: amplitude B of the slower process.

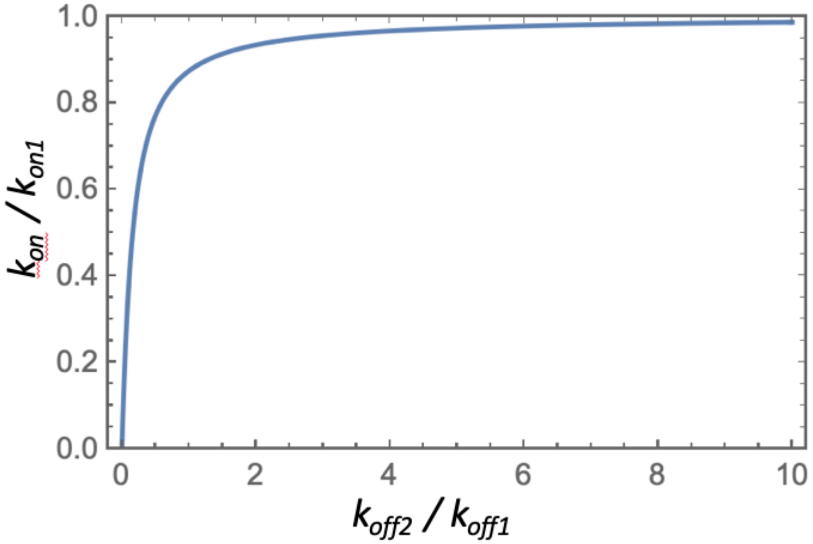

Figure S7. Dependence of k_{on} as determined in the NW model on the ratio of the off coefficients for the processes 1 and 2. The line has been calculated for K_2 = 0.3.

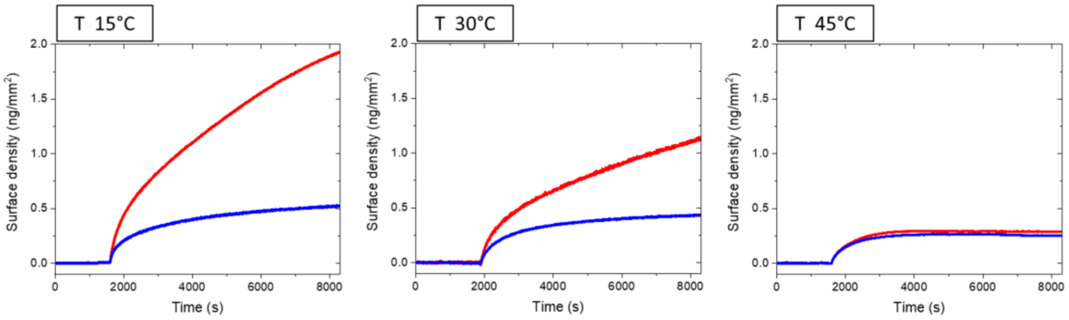

Figure S8. GAL4-DNA binding curves at different temperatures. Increase of surface density upon protein binding to specific (red) and nonspecific (blue) hairpin sequences for 50 nM concentration of GAL4 in solution at different temperatures *T*: 15°C (left), 30°C (center) and 45°C (right). All experiments were performed at 150 mM NaCl.

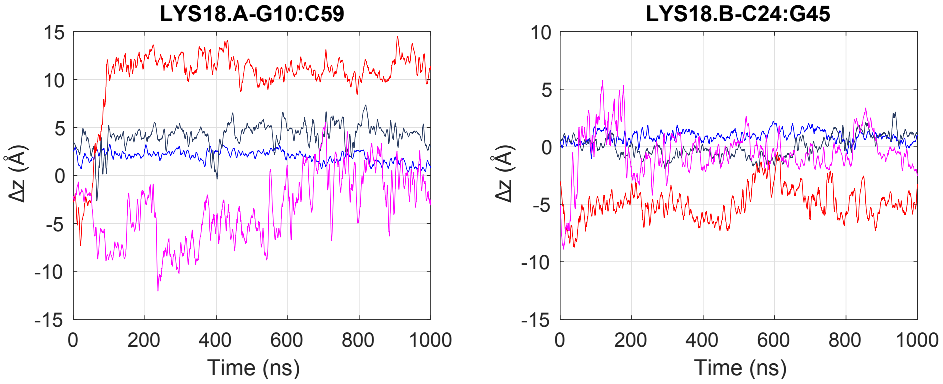

Figure S9. Displacement along the z-axis of the LYS18.A with respect to the base pair G10:C59 (left) and the LYS18.B with respect to the base pair G10:C59 (right). Blue: Gal4 with the specific DNA sequence for repeat_1. Dark blue: Gal4 with the specific DNA sequence for repeat_2. Red: Gal4 with the nonspecific DNA sequence for repeat_1. Magenta: Gal4 with the nonspecific DNA sequence for repeat_2. To sake of clarity a moving average was used.

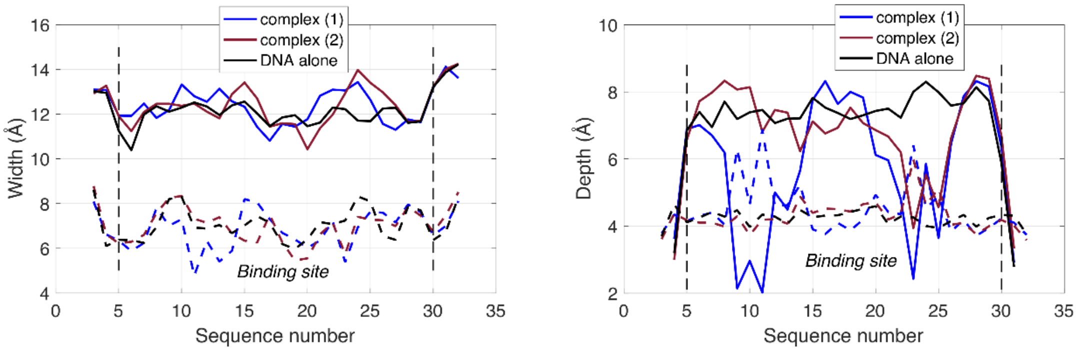

Figure S10. DNA groove dimensions (Å), width (left) and depth (right), within the isolated DNA oligomer (black lines) and within the GAL4-DNA specific complex for the first (blue lines) and second replica (dark red line). Major groove dimensions are indicated with solid lines and minor groove dimensions with dashed lines. Vertical dashed lines indicate the protein-binding site.

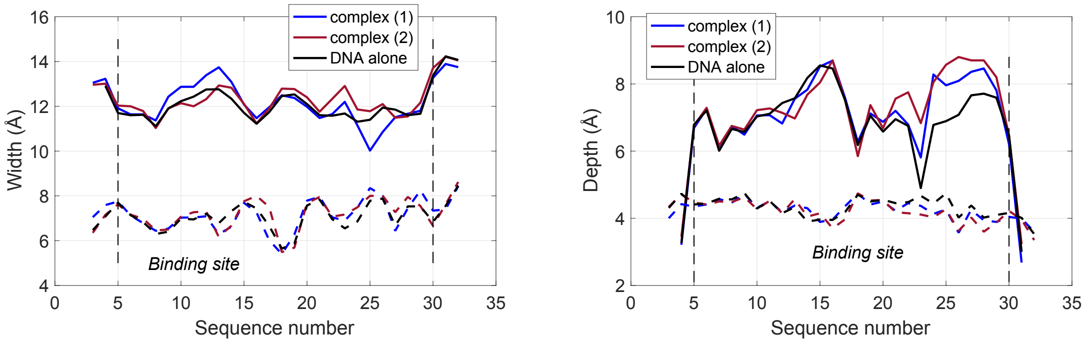

Figure S11. DNA groove dimensions (Å), width (left) and depth (right), within the isolated DNA oligomer (black lines) and within the GAL4-DNA nonspecific complex for the first (blue lines) and second replica (dark red line). Major groove dimensions are indicated with solid lines and minor groove dimensions with dashed lines. Vertical dashed lines indicate the protein-binding site.

a) b)

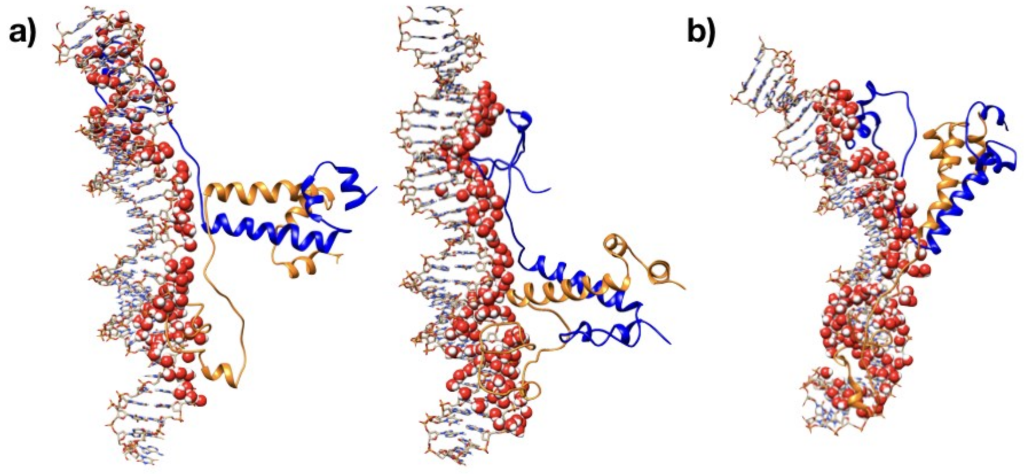

Figure S12. Water molecules within a distance of 4 Å from both the protein and the DNA sequences. a) GAL4 in complex with the nonspecific DNA sequence. b) GAL4 in complex with the specific DNA sequence.

Table S1. Additional control sequences for GAL4 binding.The red part represents the region that differs between specific and nonspecific strands. CGG and CCG sequences, important for GAL4 binding, are underlined.

Name	Length	Sequence
GAL4-HP	106	/5AmMC6/AAA AAA AAA ATG AAA TGT TGG **AAG GGT CGG AGG ACA GTC CTC CG**G GTG GTA TAG TCT CCT ACC TAT ACC ACC **CGG AGG ACT GTC CTC CGA CCC TT**C CAA CAT TTC A
NSP-HP	106	/5AmMC6/AAA AAA AAA ATG AAA TGT TGG **TTG CGT CTC TCC TAT GTT GCG TC**G GTG GTA TAG TCT CCT ACC TAT ACC ACC **GAC GCA ACA TAG GAG AGA CGC AA**C CAA CAT TTC A
CTRL-HP	98	/5AmMC6/AAA AAA AAA AGT GAA GAG GAT GTG AAA TGT TGG GAC ACT AGG TGG CAT AGT CTC CTA CCT ATG CCA CCT AGT GTC CCA ACA TTT CAC ATC CTC TTC AC
CTRL-SS	50	/5AmMC6/AAA AAA AAA AGT GAA GAG GAT GTG AAA TGT TGG GAC ACT AGG TGG CAT AG
GAL4-BE	54	/5AmMC6/AAA AAA AAA ATG AAA TGT TGG **AAG GGT CGG AGG ACA GTC CTC CG**G GTG GTA TAG
NSP-BE	54	/5AmMC6/AAA AAA AAA ATG AAA TGT TGG **TTG CGT CTC TCC TAT GTT GCG TC**G GTG GTA TAG
GAL4-BE-C	44	C TAT ACC ACC **CGG AGG ACT GTC CTC CGA CCC TT**C CAA CAT TTC A
NSP-BE-C	44	C TAT ACC ACC **GAC GCA ACA TAG GAG AGA CGC AA**C CAA CAT TTC A

Table S2. Hydrogen bond occupancy and lifetime for the specific DNA sequence.

	Res 1	Res 2	Receptor	Donor	Occupancy % (1)	Occupancy % (2)
GC	LYS20A	DG	NZ	N7	-	15.4
GC	LYS20A	DG	NZ	N7	-	23.2
	LYS20A	DG	NZ	O6	-	28.2
	LYS17A	DG	NZ	OP1	14.5	2.4*
TA	LYS17A	DT	NZ	OP2	0.4*	18.4
	LYS17A	DT	NZ	OP1	30.8	20.9
CG	DC	LEU19A	N4	O	-	15.5
	DC	LYS17A	N4	O	85.7	1.1*
GC	LYS18A	DG	NZ	O6	0.3*	13.9
	LYS18A	DG	NZ	N7	77.3	24.7
	DC	LYS	N4	O	89.2	1.2
	LYS20A	DC	NZ	OP2	13.4	0.7*
GC	LYS18A	DG	NZ	O6	65.3	32.3
	LYS23A	DC	NZ	OP2	1.7	3.5
	LYS27A	DC	NZ	OP1	-	10.5
	LYS25A	DC	NZ	OP2	-	10.8
	SER22A	DC	OG	OP2	13.4	0.2*
	LYS20A	DC	NZ	OP2	19.5	0.4*
	CYS21A	DC	N	OP1	61.7	1.3*
AT	ARG15A	DT	NH1	OP1	12.1	11.4
	ARG15A	DT	NH2	OP1	27.2	22.8
	ARG15A	DT	NH2	OP2	13.2	6.3*
	ARG15A	DT	NE	OP1	23.5	6.4*
	ALA10A	DT	N	OP2		
GC	ARG46A	DC	NH1	OP2	0.3*	12.4
	ARG46A	DC	NH2	OP2	0.7*	14.9
	LYS45A	DC	NZ	OP2	1.5*	23.9
	ARG46A	DC	NH2	OP1	0.1*	26.5
	LYS43A	DC	NZ	OP1	20.5	0.2*
	ARG15A	DC	NH2	OP1	18.3	4.9*
	ARG15A	DC	NH1	OP1	16.6	5.2*
GC	ARG46A	DC	NH1	OP2	31.7	11.5
	ARG51B	DC	NE	OP2	-	21.6
	ARG46A	DC	NH1	OP1	-	23.7
	ARG51B	DC	NE	OP1	42.1	52.5
	ARG51B	DC	NH2	OP2	7.4*	66.8
	ARG46A	DC	NH2	OP2	13.7	2.6*
AT	LYS45A	DA	NZ	N3	28.7	-
	ARG51B	DT	N	OP2	8.3*	96.7

	ARG51B	DT	NH2	OP2	13.2	-
CG	SER47B	DG	OG	OP2	0.6*	11.6
	DG	ARG46A	N2	O	34.2	-
	ARG51B	DG	N	OP2	42.1	-
AT	LYS43A	DA	NZ	OP2	-	20.9
	LYS45A	DA	NZ	OP2	13.9	-
	SER47A	DA	OG	O3'	13.8	-
	LYS45B	DT	NZ	OP2	3	13.7
GC	SER47A	DG	OG	OP2	12.1	-
TA	ARG51A	DT	N	OP2	56.9	54.9
	LYS45B	DT	NZ	O2	15.7	-
CG	ARG51A	DC	NE	OP1	9.6	1.7
	ARG46B	DC	NE	OP1	4.9*	11.6
	ARG46B	DC	NE	OP2	12.8	16.7
	ARG46B	DC	NH2	OP1	10.7	17.7
	ARG46B	DC	NH2	OP2	10.8	19.7
	SER47B	DC	N	OP2	-	30.7
	ARG46B	DC	N	OP2	11.1	38.3
	ARG51A	DC	NH1	OP2	16.6	5.8*
	ARG51A	DC	NH2	OP2	14.4	6.6*
CG	LYS45B	DC	NZ	OP2	4.6*	23.3
	ARG46B	DC	NH2	OP1	11.9	0.4*
TA	GLU8B	DT	N	OP2	-	17.2
	GLN9B	DT	NE2	OP1	-	18.4
	LYS43B	DT	NZ	OP2	29.1	0.6*
	ALA10B	DT	N	OP2	12.8	5.0*
	ARG15B	DT	NE	OP1	21	5.6*
	ARG15B	DT	NH2	OP1	27.3	6.0*
	ARG15B	DT	NH2	OP2	15.1	6.0*
CG	GLN9B	DC	NE2	OP1	-	17
	CYS21B	DC	N	OP1	0.1*	24.4
	DC	ARG15B	N4	O	10.3	-
	CYS21B	DC	N	O3’	1.6	-
	LYS25B	DC	NZ	OP1	33.8	-
	LYS25B	DC	NZ	OP2	25.2	-
	LYS18B	DG	NZ	O6	51.2	50.2
CG	LYS20B	DC	NZ	OP2	-	10.3
	DC	LYS18B	N4	O	87.3	54.1
	CYS21B	DC	N	OP1	68.6	-
	LYS18B	DG	NZ	O6	4.3*	12.3
	LYS178B	DG	NZ	N7	52.9	56.9
GC	LYS20B	DG	NZ	N7	19.3	0.3*

	DC	LYS17B	N4	O	34	43
GC	LYS20B	DG	NZ	N7	10.9	5.6*
	LYS17B	DC	NZ	OP1	21.7	14.7
	DC	LYS18B	N4	O	0.2*	23.7
GC	LYS20B	DG	NZ	N7	-	11.2
	LYS17B	DC	NZ	OP1	27.9	22.3
TA	LYS30B	DA	NZ	OP2	23.9	4.3

Red: H-bonds found only in the MD simulations. Black: H-bonds found both in the x-ray and the MD simulations. Blue: H-bonds found only in the starting structure. Only HBs observed in MD simulations with an occupancy > 10% are shown. *: HBs with an occupancy < 10% in one repeat or in the case they were found in the x-ray and the MD simulations.

Table S3. Hydrogen bond occupancy and lifetime for the nonspecific DNA sequence.

	Res 1	Res 2	Receptor	Donor	Occupancy % (1)	Occupancy % (2)
GC	LYS17A	DG	NZ	OP2	0.1*	1.0*
TA	LYS36A	DT	NZ	OP2	15.9	-
CG	LYS30A	DC	NZ	OP1	21.3	0.2*
	LYS17A	DC	NZ	OP1	11.1	-
TA	LYS17A	DT	NZ	OP1	14.7	-
CG	LYS20A	DG	NZ	N7	12.5	-
	LYS23A	DG	NZ	OP2	0.1*	5.0*
TA	LYS18A	DT	NZ	O4	15.2	-
	ARG15A	DA	NH1	OP1	1.1*	46.1
	GLN9A	DA	NE2	OP2	0.5*	19
CG	ARG15A	DG	NH2	OP2	-	56.7
	LYS18A	DG	NZ	O6	33.9	-
	ARG15A	DG	NH1	O5’	-	32.1
	LYS23A	DG	NZ	OP2	19.3	-
	ARG15A	DG	NH1	OP1	-	18.3
	ARG39A	DG	NE	OP1	-	17.7
	ARG39A	DG	NH2	OP1	-	14.3
CG	ARG15A	DG	NE	OP1	40.7	-
	ARG15A	DG	NH2	OP2	29.8	-
	LYS23A	DG	NZ	OP2	28.1	-
	ARG46A	DG	NH1	OP2	0.2*	27.6
	ARG15A	DG	NH2	OP1	25.5	-
	ARG39A	DG	NE	OP1	-	25.3
	ARG39A	DG	NH2	OP1	-	18.7
	LYS18A	DG	NZ	O6	14.4	-
	LYS45A	DG	NZ	N3	-	12.1
	LYS18A	DG	NZ	N7	12	-
	ARG46A	DG	NE	OP2	-	10.7
	ARG51B	DG	NH2	OP2	1.9*	5.2*
TA	ARG51B	DA	N	OP2	-	64.1
	LYS45A	DA	NZ	OP2	12.6	-
	ARG46A	DA	NH2	O3’	-	11.2
AT	LYS43A	DT	NZ	OP2	12.9	-
TA	ARG15B	DT	NH2	OP2	-	12.6
	ARG46B	DT	NH2	OP2	0.5*	1.5*
GC	ARG15B	DG	NH1	OP1	8.9*	41.2
	ARG46B	DG	NH2	OP2	-	26.9

	ALA10B	DG	N	OP2	-	25.6
	LYS18B	DG	NZ	O6	0.1*	24.9
	ARG15B	DG	NE	OP1	0.5*	22.1
	ARG15B	DG	NH2	OP1	8.6*	20.7
	ARG46B	DG	NH1	OP2	-	18.3
	ARG15B	DG	NH2	OP2	5.6*	17.6
	LYS18B	DG	NZ	OP1	14.6	-
GC	LYS20B	DG	NZ	O6	22.1	6.1*
	LYS25B	DG	NZ	OP2	-	13.5
	LYS23B	DG	N	OP1	-	13.5
	LYS18B	DG	NZ	N7	1.5*	13.5
TA	SER22B	DT	N	OP1	-	12.6
	LYS20B	DT	NZ	O4	22.1	8.7*
	LYS20B	DT	NZ	OP1	-	17.1
	LYS18B	DT	NZ	O4	0.4*	14.2
	LYS23B	DT	NZ	OP2	-	13
	ARG46B	DA	NH1	OP2	14.3	-
CG	LYS17B	DG	NZ	OP1	36	0.9*
	LYS20B	DG	NZ	O6	11.9	7.9*
GC	LYS20B	DG	NZ	O6	-	22.1
	LYS20B	DG	NZ	N7	-	11.2
	LYS17B	DC	NZ	OP2	23.7	1.2*
	LYS17B	DC	NZ	OP1	23.1	18.7
	LYS17B	DC	NZ	O5'	17.2	0.1*
	LYS45B	DC	NZ	OP2	16.1	-
GC	LYS20B	DG	NZ	O6	-	19
	LYS17B	DC	NZ	OP1	-	23.3
	LYS17B	DC	NZ	OP2	-	10.1
TA	SER22B	DA	OG	OP2	24.9	-
	LYS30B	DA	NZ	OP2	15.6	11.6
GC	LYS43B	DG	NZ	OP2	18.3	-
	LYS30B	DC	NZ	OP2	17.4	2.1*
	LYS33B	DC	NZ	OP2	1.0*	10.4
GC	ARG39B	DG	NH2	OP2	22.7	-
	DG	ASN34B	N2	OD1	10.8	-
	ARG39B	DG	NH1	OP2	10.6	-
TA	ARG39B	DT	NH1	OP2	27.9	-
	ARG39B	DT	NH2	OP2	18.9	-
AT	LYS33B	DA	NZ	OP2	13.1	-

Red: H-bonds found only in the MD simulations. Black: H-bonds found both in the x-ray and the MD simulations. Blue: H-bonds found only in the starting structure. Only HBs observed in MD simulations with an occupancy > 10% are shown. *: HBs with an occupancy < 10% in one repeat or in the case they were found in the x-ray and the MD simulations.

Table S4. Decomposition of the entropic terms from MD simulations.

<table>
<tr><th></th><th>Specific (cal mol^{-1} K^{-1})</th><th>Nonspecific (cal mol^{-1} K^{-1})</th></tr>
<tr><td>$\langle S_{complex} \rangle$</td><td>15 657</td><td>15 787</td></tr>
<tr><td>$\langle S_{DNA,\ complex} \rangle$</td><td>4258</td><td>4226</td></tr>
<tr><td>$\langle S_{protein,\ complex} \rangle$</td><td>11 545</td><td>11 695</td></tr>
<tr><td>$\Delta S_{protein/DNA,complex}$</td><td>146</td><td>134</td></tr>
<tr><td>$\langle S_{DNA,unbound} \rangle$</td><td>4 334</td><td>4 319</td></tr>
<tr><td>ΔS_{DNA}</td><td>-76</td><td>-93</td></tr>
<tr><td>$S_{protein,unbound}$</td><td colspan="2">11 412</td></tr>
<tr><td>$\Delta S_{protein}$</td><td>133</td><td>283</td></tr>
</table>

Table S5. Energetic terms involved in the decomposition of binding energy from MD simulations.

	**Specific** (kcal/mol)	**Nonspecific** (kcal/mol)
$\Delta E_{el} + \Delta G_{ps}$ (ε=4)	-17±10	-2±31
ΔG_{np}	-12 ±3	-11.6±0.3
ΔG_{NP}=ΔG_{np} + ΔE_{vdw}	-69±28	-49±14
-TΔS	26±2	-17±14
$\Delta G_{binding}$	-60 ± 30	-68±37

E_{vdw}: the molecular mechanics van der Waals energy contribution. G_{ps}: polar contribution to the solvation energy. G_{el}: molecular mechanics electrostatic energy. G_{np}: the non-polar solvation energy. -TS_s: the solute entropy.

References

1. Bulyk, M. L., Huang, X., Choo, Y., and Church, G. M. (2001) Exploring the DNA-binding specificities of zinc fingers with DNA microarrays. *Proc. Natl. Acad. Sci.* **98**, 7158–7163
2. Nutiu, R., Friedman, R. C., Luo, S., Khrebtukova, I., Silva, D., Li, R., Zhang, L., Schroth, G. P., and Burge, C. B. (2011) Direct measurement of DNA affinity landscapes on a high-throughput sequencing instrument. *Nat. Biotechnol.* **29**, 659–664
3. Shumaker-Parry, J. S., Aebersold, R., and Campbell, C. T. (2004) Parallel, Quantitative Measurement of Protein Binding to a 120-Element Double-Stranded DNA Array in Real Time Using Surface Plasmon Resonance Microscopy. *Anal. Chem.* **76**, 2071–2082
4. Salina, M., Giavazzi, F., Lanfranco, R., Ceccarello, E., Sola, L., Chiari, M., Chini, B., Cerbino, R., Bellini, T., and Buscaglia, M. (2015) Multi-spot, label-free immunoassay on reflectionless glass. *Biosens. Bioelectron.* **74**, 539–545
5. Nava, G., Ceccarello, E., Giavazzi, F., Salina, M., Damin, F., Chiari, M., Buscaglia, M., Bellini, T., and Zanchetta, G. (2016) Label-free detection of DNA single-base mismatches using a simple reflectance-based optical technique. *Phys. Chem. Chem. Phys.* **18**, 13395–13402
6. Vörös, J. (2004) The density and refractive index of adsorbing protein layers. *Biophys. J.* **87**, 553–561
7. Marmorstein, R., Carey, M., Ptashne, M., and Harrison, S. C. (1992) DNA recognition by GAL4: structure of a protein-DNA complex. *Nature*. **356**, 408–414
8. Lavery, R., Zakrzewska, K., and Sklenar, H. (1995) JUMNA (junction minimisation of nucleic acids). *Comput. Phys. Commun.* **91**, 135–158
9. Pérez, A., Marchán, I., Svozil, D., Sponer, J., Cheatham, T. E., Laughton, C. A., and Orozco, M. (2007) Refinement of the AMBER force field for nucleic acids: Improving the description of α/γ conformers. *Biophys. J.* **92**, 3817–3829
10. Tsui, V., and Case, D. A. (2000) Theory and applications of the Generalized Born solvation model in macromolecular simulations. *Biopolymers*. **56**, 275–291
11. Pronk, S., Páll, S., Schulz, R., Larsson, P., Bjelkmar, P., Apostolov, R., Shirts, M. R., Smith, J. C., Kasson, P. M., Van Der Spoel, D., Hess, B., and Lindahl, E. (2013) GROMACS 4.5: A high-throughput and highly parallel open source molecular simulation toolkit. *Bioinformatics*. **29**, 845–854
12. Lindorff-Larsen, K., Piana, S., Palmo, K., Maragakis, P., Klepeis, J. L., Dror, R. O., and Shaw, D. E. (2010) Improved side-chain torsion potentials for the Amber ff99SB protein force field. *Proteins Struct. Funct. Bioinforma.* **78**, 1950–1958
13. Horn, H. W., Swope, W. C., and Pitera, J. W. (2005) Characterization of the TIP4P-Ew water model: Vapor pressure and boiling point. *J. Chem. Phys.* 10.1063/1.2085031
14. Joung, I. S., and Cheatham, T. E. (2008) Determination of alkali and halide monovalent ion parameters for use in explicitly solvated biomolecular simulations. *J. Phys. Chem. B*. **112**, 9020–9041
15. Pang, Y.-P. (1999) Novel Zinc Protein Molecular Dynamics Simulations: Steps Toward Antiangiogenesis for Cancer Treatment. *J. Mol. Model.* **5**, 196–202
16. Berendsen, H. J. C., Postma, J. P. M., Van Gunsteren, W. F., Dinola, A., and Haak, J. R. (1984) Molecular dynamics with coupling to an external bath. *J. Chem. Phys.* **81**, 3684–3690
17. Bussi, G., Donadio, D., and Parrinello, M. (2007) Canonical sampling through velocity rescaling. *J. Chem. Phys.* 10.1063/1.2408420
18. Parrinello, M., and Rahman, A. (1981) Polymorphic transitions in single crystals: A new molecular dynamics method. *J. Appl. Phys.* **52**, 7182–7190
19. Lavery, R., Moakher, M., Maddocks, J. H., Petkeviciute, D., and Zakrzewska, K. (2009) Conformational analysis of nucleic acids revisited: Curves+. *Nucleic Acids Res.* **37**, 5917–5929
20. McGibbon, R. T., Beauchamp, K. A., Harrigan, M. P., Klein, C., Swails, J. M., Hernández, C. X., Schwantes, C. R., Wang, L. P., Lane, T. J., and Pande, V. S. (2015) MDTraj: A Modern Open Library for the Analysis of Molecular Dynamics Trajectories. *Biophys. J.* **109**, 1528–1532

21. Kollman, P. (1993) Free energy calculations: applications to chemical and biochemical phenomena. *Chem. Rev.* **93**, 2395–2417
22. Gilson, M. K., Sharp, K. A., and Honig, B. H. (1988) Calculating the electrostatic potential of molecules in solution: Method and error assessment. *J. Comput. Chem.* **9**, 327–335
23. Zhou, Y. C., Feig, M., and Wei, G. W. (2008) Highly accurate biomolecular electrostatics in continuum dielectric environments. *J. Comput. Chem.* **29**, 87–97
24. Li, L., Li, C., Sarkar, S., Zhang, J., Witham, S., Zhang, Z., Wang, L., Smith, N., Petukh, M., and Alexov, E. (2012) DelPhi: a comprehensive suite for DelPhi software and associated resources. *BMC Biophys.* **5**, 9
25. Frishman, D., and Argos, P. (1995) Knowledge-based protein secondary structure assignment. *Proteins Struct. Funct. Bioinforma.* 10.1002/prot.340230412
26. Furini, S., Barbini, P., and Domene, C. (2013) DNA-recognition process described by MD simulations of the lactose repressor protein on a specific and a non-specific DNA sequence. *Nucleic Acids Res.* **41**, 3963–3972
27. Wang, W., and Kollman, P. A. (2000) Free energy calculations on dimer stability of the HIV protease using molecular dynamics and a continuum solvent model. *J. Mol. Biol.* **303**, 567–582
28. Yu, Y. B., Privalov, P. L., and Hodges, R. S. (2001) Contribution of translational and rotational motions to molecular association in aqueous solution. *Biophys J*. **81**, 1632–1642
29. Spolar, R. S., and Record, M. T. (1994) Coupling of local folding to site-specific binding of proteins to DNA. *Science (80-.).* 10.1126/science.8303294
30. Page, M. I., and Jencks, W. P. (1971) Entropic contributions to rate accelerations in enzymic and intramolecular reactions and the chelate effect. *Proc Natl Acad Sci U S A*. **68**, 1678–1683
31. Schlitter, J. (1993) Estimation of absolute and relative entropies of macromolecules using the covariance matrix. *Chem. Phys. Lett.* **215**, 617–621
32. Hess, B. (2002) Determining the shear viscosity of model liquids from molecular dynamics simulations. *J. Chem. Phys.* **116**, 209
33. Hou, T., Wang, J., Li, Y., and Wang, W. (2011) Assessing the Performance of the MM/PBSA and MM/GBSA Methods. 1. The Accuracy of Binding Free Energy Calculations Based on Molecular Dynamics Simulations. *J. Chem. Inf. Model.* **51**, 69–82
